# The Role of Animal Assisted Intervention on Improving Self-Esteem in Children With Attention Deficit/Hyperactivity Disorder

**DOI:** 10.3389/fped.2018.00300

**Published:** 2018-11-02

**Authors:** Sabrina E. B. Schuck, Heather L. Johnson, Maryam M. Abdullah, Annamarie Stehli, Aubrey H. Fine, Kimberley D. Lakes

**Affiliations:** ^1^Pediatrics, School of Medicine, University of California, Irvine, Irvine, CA, United States; ^2^School Psychology, College of Education, University of Utah, Salt Lake City, UT, United States; ^3^Greater Good Science Center, University of California, Berkeley, Berkeley, CA, United States; ^4^Education, California State Polytechnic University, Pomona, CA, United States; ^5^Psychiatry, School of Medicine, University of California, Riverside, Riverside, CA, United States

**Keywords:** Human Animal Interaction, Animal Assisted Intervention, therapy dogs, ADHD, self-esteem, self-awareness, school-based interventions, social skills training

## Abstract

Attention Deficit/Hyperactivity Disorder (ADHD), the most ubiquitous mental health problem in children, has been associated with poor self-esteem. Psychosocial interventions have aimed to improve self-esteem among this group, with the aim of reducing the development of comorbid depression and anxiety. The present study implemented a randomized control design to examine the possibility of Animal Assisted Interventions (AAI) as a viable approach to improving self-esteem among children with ADHD. Children's self-esteem across multiple domains as measured by the Self-Perception Profile for Children was evaluated (*n* = 80, ages 7–9, 71% male). To test the hypothesis that AAI improves self-esteem, stratified Wilcoxon Signed-Rank Tests (SAS NPAR1WAY procedure) were used to compare pre- to post-treatment ratings. Analyses indicated that scores of children's self-perceptions in the domains of behavioral conduct, social, and scholastic competence, were significantly increased from baseline to post-treatment in the AAI group (*z* = 2.320, *p* = .021, *z* = 2.631, *p* = .008, and *z* = 2.541, *p* = .011, respectively), whereas pre-post-treatment differences in self-perceptions were not found for the children in the control group without AAI. Findings suggest that AAI is a viable strategy for improving ratings of self-perceived self-esteem in children with ADHD.

## Introduction

Attention Deficit/Hyperactivity Disorder (ADHD) is the most prevalent mental health disorder of childhood and despite intervention continues to impair individuals across the lifespan when compared to their typically developing peers (1). Children with ADHD are characteristically impaired by deficits in skills of Executive Function (EF) including attention, working memory, and inhibition—all skills essential to self-awareness and self-regulation. Children with ADHD oftentimes present with associated poor social skills and/or problem behaviors and are at greater risk for developing other mental health disorders by young adulthood (2). Pharmacotherapy (e.g., atomoxetine, methylphenidate) is the mainstay of traditional medical intervention for ADHD, but treatment failures are common (3), there is evidence of slowed growth in individuals who take stimulants for long periods of time (4, 5) and parents of young children are increasingly seeking alternatives to medication treatments. Of note, many parents find alternative therapies, including Animal Assisted Intervention (AAI), to be more acceptable than medication (6).

Early on it was hypothesized that Human Animal Interaction (HAI) and AAI with children contributed to improvements on measures of psychological well-being, such as self-esteem (7, 8) or closely related constructs (9, 10), while others reported no AAI impact (11, 12). The inconsistent findings of these early studies seems likely to have been the product of weak research designs.

Reviews of HAI and AAI indicate an abundance of correlational, hypothesis-generating studies (13–16) and conclude that the field would be further strengthened by rigorous experimental, evidence-based intervention designs (17, 18). While groundbreaking in hypotheses generation, most AAI research has been criticized for the lack of control conditions and specificity of intervention aims (19). Many of these studies describe solely correlational findings. Perhaps, one of most problematic areas of AAI research, plagued by both of these weaknesses, is the examination of the relationship of AAI and self-esteem—a nebulous construct to start with.

The concept of self-esteem has been defined as “the level of global regard that one has for the self as a person” [(20), p. 88]. In typically developing populations, self-esteem has been linked to task persistence, achievement and overall outcomes. Low self-esteem has been linked to poor outcomes, depression and other mental health disorders (20). Considering the frequent adverse social feedback that children with ADHD are likely to experience throughout their development, it seems plausible that these experiences may contribute to low self-esteem.

It is clear that children with ADHD are at greater risk for poor outcomes and the development of comorbid mental health disorders (21). The role of self-esteem in predicting these outcomes is less understood (22). Previous research has examined the role of self-esteem in prognosis and the later development of comorbid mental health disorders in young adulthood (23). Historically, psychosocial interventions have targeted increased self-esteem in children with ADHD (24), with the aim of reducing the development of comorbid depression and anxiety.

Positive Assertive Cooperative Kids (Project P.A.C.K.), a randomized controlled trial, was designed to examine the safety and efficacy of AAI with dogs vs. traditional psychosocial treatment strategies for children with ADHD (25). The P.A.C.K. model reduced symptoms of ADHD and problem behaviors and improved social skills (26). A secondary aim of Project P.A.C.K. was to determine if AAI contributed to an increase in self-esteem as measured by children's self-perceptions of their competence across multiple domains. We hypothesized that the children participating in the therapy with dogs would have greater gains in their self-esteem than those children participating in therapy as usual.

## Methods

### Participants

This study was approved by the local university Institutional Review Board. Written and informed consent was obtained from parents and written and informed assent was obtained from child participants. Additionally, upon review by the local Institutional Animal Care and Use Committee (IACUC), this study was determined exempt from IACUC review as the participation of the therapy animals was not outside the scope of their normal activity and because they were not the subject of investigation. Eighty-eight children with ADHD, ages 7–9 years, (71% male), and their parents participated in Project P.A.C.K., a randomized controlled trial examining the safety and efficacy of traditional “best practice” psychosocial intervention with and without therapy dogs, 82 completed treatment across 12 weeks and participated in a follow up assessment 6 weeks later (26). Eighty children and families completed the measures included in this analysis at baseline, end of treatment and at follow-up.

#### Screening and eligibility criteria

Participants were selected using a multi-gate screening procedure to determine eligibility. Parents completed a family medical and psychosocial history questionnaire. Researchers administered the Kaufman-Schedule for Affective Disorders and Schizophrenia for School-Age Children: Present and Lifetime Version [K-SADS-PL; (27)], a semi-structured clinician-administered interview conducted with parents and children, based on criterion set forth in the Diagnostic and Statistical Manual of Mental Disorders [4th ed., text rev.; DSM-IV- TR; (28)] for psychiatric disorders. Children also completed the Wechsler Abbreviated Scale of Intelligence, Second Edition [WASI-II; (29, 30)]. Eligibility criteria included a primary diagnosis of ADHD, Combined subtype, aged 7–9 years, an estimated full scale IQ score of 80 or above, and the ability to complete all screening measures. Exclusionary criteria included current use of medication for ADHD; a diagnosis of a pervasive developmental disorder/autism, depression, anxiety, or epilepsy; and a history of cruelty to animals.

#### Randomization design

All participants were randomly assigned to one of two treatment groups: (a) a cognitive-behavioral group therapy incorporating a AAI with therapy dogs/handler dyads or (b) a cognitive-behavioral group therapy without therapy dogs (non-AAI). In efforts to establish treatment efficacy for both the AAI and the non-AAI treatment groups, a waitlist condition was implemented to control for the possible influence of time and child development on symptom severity in both groups. Specifically, half of all recruited participants, regardless of treatment group, were consented and assessed and then experienced a waiting period of 12 weeks prior to a subsequent assessment and the start of treatment. The remainder of participants recruited began immediate treatment (IT) subsequent to consent and assessment.

### Measures

Children participated in a battery of screening measures, including a brief assessment of cognitive skills, at intake prior to intervention (described above). Subsequent to screening and randomization, they participated in direct assessment which included measures of performance, self-evaluations and structured interviews in the context of a laboratory school setting at three time points: (a) immediately prior to, (b) immediately following, and (c) 6 weeks after the 12-week intervention period. Simultaneously, parents rated their children's social skills competence, severity of ADHD symptoms, and problem behaviors.

#### Intelligence

The Wechsler Abbreviated Scale of Intelligence, Second Edition [WASI-II; (29, 30)] is a brief measure utilizing four subtests with the highest factor loading on generalized intelligence (g). The administration takes about 3 min and yields a Verbal Comprehension Index (VCI; Vocabulary and Similarities), a Perceptual Reasoning Index (PRI; Block Design and Matrix Reasoning) and an estimated Full Scale IQ (FSIQ-4). Cronbach's alpha coefficients for all subscales of this measure are high, ranging from the high 0.80s to 0.99, indicating good to excellent reliability in child samples, and the measures has demonstrated acceptable to excellent validity with other established measures utilized for estimating *g* (31). Participants completed the WASI at baseline, no more than 2 weeks prior to participation in the laboratory school day assessments and the intervention phase.

#### Self-esteem

The Self-Perception Profile for Children [SPPC; (32)] is a 36-item scale measuring perceived global self-worth in children. Subscales in this measure include scholastic competence, social acceptance, athletic competence, physical appearance, and behavioral conduct, and a cumulative measure of global self-worth. Cronbach's alpha coefficients for each domain exceed 0.80, indicating acceptable to excellent reliability and validity testing, resulting in clear factor loadings with basic oblique rotation-five loadings, ranged between 0.41 and 0.90 as reported in the most recent update to the SPPC manual (32). This scale was administered to child participants individually by a trained research assistant who read the items aloud in keeping with recommendations in the most recent manual for children younger than Grade 5. Of note, in consideration of the attention challenges of the special population the scale was administered with the aid of a pictorial visual analog to assist the child in choosing their rating. The measure was completed on the Saturdays immediately preceding and following the intervention period, and then at a 6-week follow-up in the context of the laboratory school day setting.

#### Social skills and problem behaviors

Social Skills Improvement System-Parent Form [SSIS-P; (33)] is a 79-item scale measuring social skills and problem behaviors in children as reported by their parents. In the Social Skills domain, the subscales are communication, cooperation, assertion, responsibility, empathy, engagement, and self-control. Subscales in the Problem Behaviors domain include internalizing, externalizing, bullying, hyperactivity/inattention, and autism spectrum. Gresham and Elliott (33) provide an extensive psychometric review of the SSIS in the administration manual and report reliability tests for Cronbach's alpha coefficients for the main domains all exceeding 0.90 indicating very good to extremely high reliability for children between the ages of 5–12, with measures of validity reported in the moderate to high range (pp. 65–136). Commensurate with their child's participation in each of the laboratory school days, one parent respondent (91% female) rated their child's social skills and problem behaviors, as measured by the SSIS.

#### ADHD and ODD symptoms

The Attention-Deficit/Hyperactivity Disorder Rating Scale, Fourth Edition [ADHD-RS-IV; (34)] is an established measure of ADHD symptoms derived from the Diagnostic and Statistical Manual of Mental Disorders 4th Edition [DSM-IV; (28)] with Cronbach's alpha coefficients all exceeding 0.85, indicating extremely high to excellent subscale reliability (34). In addition to the ADHD-RS items, in a similar fashion, parents rated their children on the nine symptoms of Oppositional Defiant Disorder as listed in the DSM-IV. The ADHD-RS and symptoms of ODD scale were completed by parents every 2 weeks during the course of the intervention.

#### Permanent product math test

The Permanent Product Measure of Performance [PERM-P; (35)] is a validated, time-sensitive, skill-adjusted test. The assessment is comprised of simple math problems which are required to be completed at multiple time points throughout the simulated classroom sessions of the laboratory school days. This measure has been used extensively in clinical trials examining the effects of pharmaceuticals for children with ADHD, and has been found to be a robust, objective measure of the ability to initiate a task, self-monitor/stay on task, and complete written seatwork (35).

### Intervention

For a period of 12 weeks, each child participant attended an intervention group session twice a week; 1 weekday evening for 2 hours and on Saturday for 2 $\frac{1}{2}$ hours, resulting in a total of 4 $\frac{1}{2}$ hours per week of treatment for the child. Parents received 2 hours of group-based behavioral parent training (BPT) once a week that occurred during their child's weekly evening sessions. The P.A.C.K. intervention curriculum implemented for both groups, incorporated strategies based on components from the University of California, Irvine Child Development Center School-based Social Skills model, the Kids Interacting with Dogs Safely program developed by Jane Deming and the American Humane Association (2009), and the Intermountain Therapy Animals' Reading Education Assistance Dogs program (ITA R.E.A.D® Handbook, 2003–2004).

**Figure 1:**
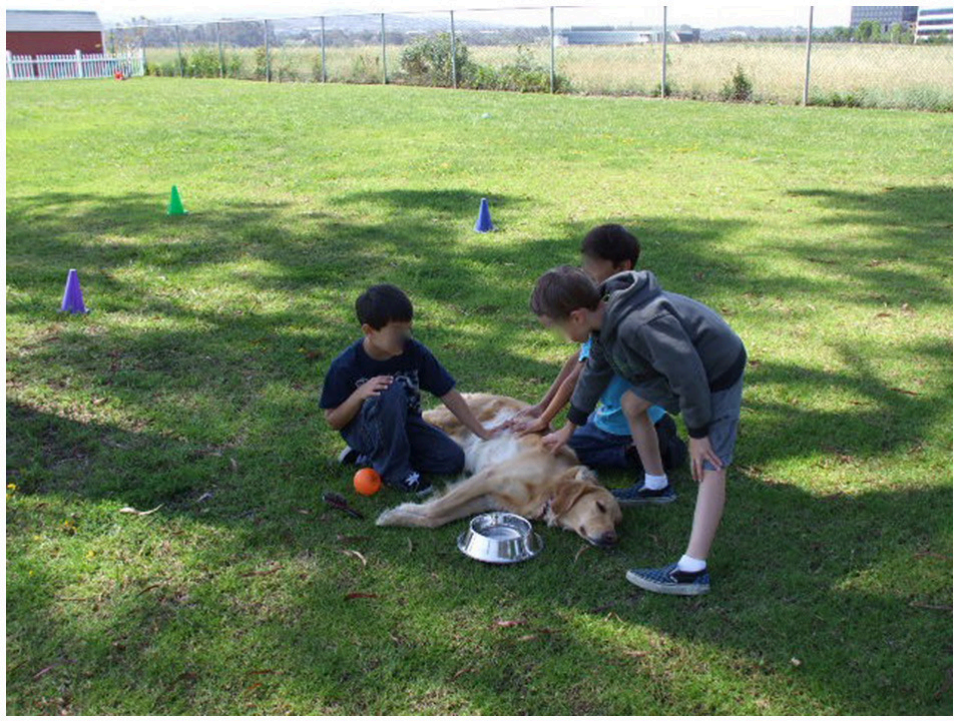
Animal Assisted Intervention with certified therapy dogs.

**Figure 2:**
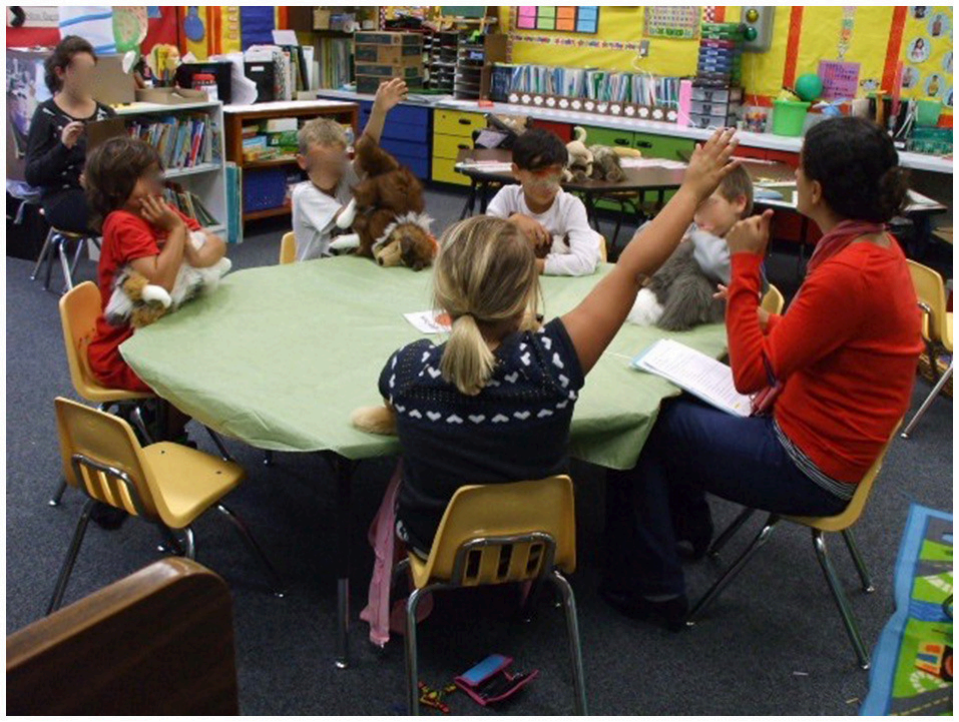
Traditional psychosocial skills training with a dog theme.

The AAI group included the participation of three certified therapy dogs, facilitated by their handlers (partners), during each intervention session (see Figure 1). The non-AAI group received the same standard treatment curriculum, but utilized toy dogs (realistic puppets/stuffed plush toys) in lieu of live dogs (see Figure 2). Of note, written and informed consent for the release of photographic images was obtained from parents of participants in these photos but the faces of the minors are masked to protect their privacy.

#### P.A.C.K. curriculum

The social skills curriculum that was implemented in each of the children's therapeutic group sessions was originally developed for the UC Irvine Child Development Center School Program, a laboratory school environment for children with ADHD. This model combines strategies based in learning and cognitive-behavioral theories and is aimed to promote adaptive skill acquisition and thereby reduce problem behaviors (36). This curriculum, along with the curriculum of the Summer Treatment Program developed by Pelham et al. (37) contributed to the psychosocial treatment strategies implemented and tested in the Multi-modal Treatment Study of ADHD (MTA Study), the largest NIH funded longitudinal study of treatment modalities for children with ADHD (38–40). These traditional evidence-based components were complemented with novel strategies (*The How to Be a Good Teacher* lessons and the *How Did I Do?* self-assessment) which aimed to increase self-esteem by developing self-competence and self-efficacy [see (25, 26)]. These lessons were inspired by a theoretical premise that interaction with dogs provides a naturalist, non-verbal, ‘feedback’ mechanism unique to AAI. The lessons were administered with all children, with and without the assistance of certified therapy dogs and puppies in training for *Canine Companions for Independence*. In the AAI group, children were supported in ‘training’ basic commands (come, sit, stay) with certified therapy dogs for the first 9 weeks of the intervention and then with the puppies in the last 3 weeks of the intervention. In the group without dogs, children were instructed and coached to teach their peers how to complete a skill or inform the peers about a specific subject of interest. See the chapter by Gee et al. (41) for a more detailed description of the specific strategies implemented.

#### Behavioral parent training (BPT)

The parent training component of intervention consisted of 12, weekly, 2-hour long sessions of BPT conducted with six families per treatment group. Sessions were based on a traditional BPT curriculum and adapted from the MTA study (40). Specifically, parents were taught behavior modification techniques and standard directive parenting strategies (e.g., giving effective directions, transitional warnings, problem solving) and how to teach self-regulation strategies, facilitate anger management, and target social skills development for their children. Parent/child shared homework activities (e.g., reading a short dog-themed story together) were assigned to encourage discussions focusing on targeted social skills and/or humane education topics.

### Analysis

To test the hypothesis that AAI increases self-esteem as measured by child self-perceptions of competence, and in consideration of the non-normal distribution of scores on the SPPC for the sample, stratified Wilcoxon Signed-Rank Tests (SAS NPAR1WAY procedure) were used. This test allows for a comparison of pre- to post- differences in a non-normal distribution and results in a z score statistic that can then be tested for significance. Considering the nature of the ranking test, a final transformation of the scores was utilized in efforts to best represent the direction of change for those individuals that had higher (improved) scores of self-perception at end of treatment when compared to their scores at baseline.

Stratified correlation analyses were performed to examine the relationship between SPPC ratings and baseline measures including: ADHD-RS subscales (ADHD, Hyperactivity, Inattention), ODD symptoms, Parent rated SSIS, the WASI (Block Design, Matrix Reasoning, Vocabulary and Similarities, Full Scale IQ), and PERM-P level.

## Results

### Prior to intervention

At baseline, groups were equivalent on self-ratings of each domain of self-esteem as measured by the SPPC, with the exception of the children in the non-AAI group rating themselves more favorably in the domain of physical appearance (*z* = 4.562, *p* < .001) and somewhat but not significantly more favorably in athletic competence (*z* = 1.951, *p* = .052). Of note, the majority of children across groups rated their competence fairly favorably, resulting in a non-normal, negatively skewed or “J-shaped” distribution of scores both before and after treatment for both groups. But, for the most part, mean scores for this particular sample did not differ significantly from published means for their typically developing, same age peers, with the exception of this sample rating themselves higher in the domain of physical appearance at baseline (non-AAI *z* = 11.441, *p* < .001; AAI *z* = 9.112, *p* < .001). As previously reported in an analysis testing equivalency of groups, randomization resulted in no significant differences in demographic characteristics or behavior skills as measured by; parent ratings on the ADHD-RS, for each subtype (Inattention, H/I, and Combined type) as well as symptoms of ODD) (26). Similarly, at baseline no group differences were revealed for cognitive skills, as measured by the WASI-II (Vocabulary, Block Design, Similarities, and Matrix Reasoning) (Table 1) or academic skills as measured by the PERM-P (χ^2^ = .05, *p* = .974).

**Table 1:** Participant Cognitive Skills by Intervention Group.

**Measure**	**Non-AAI (*n* = 41)**	**AAI (*n* = 41)**	
	***M (SD)***	***M (*SD*)***	***t***	***p***
WASI-II Vocabulary	56.27 (9.88)	53.54 (10.07)	1.24	.228
WASI-II Block Design	53.73 (10.23)	52.51 (11.16)	0.52	.607
WASI-II Similarities	60.22 (8.34)	56.56 (11.30)	1.67	.100
WASI-II Matrix Reasoning	55.81 (9.53)	55.44 (9.04)	0.18	.859
WASI-II VIQ	113.90 (14.34)	108.60 (16.62)	1.52	.132
WASI-II PIQ	108.00 (14.38)	106.90 (15.40)	0.33	.745
WASI-II FSIQ	112.40 (13.51)	108.90 (15.92)	1.08	.285

*AAI = Animal Assisted Intervention; WASI-II = Wechsler Abbreviated Scale of Intelligence, Second Edition; VI = Verbal Intelligence Quotient, PIQ = Performance Intelligence Quotient, FSIQ = Full Scale Intelligence Quotient*.

### Post intervention

As previously reported, all children who participated in the P.A.C.K. study demonstrated significant improvement in symptoms of ADHD and social skills but improvements were significantly greater in the group that participated in AAI (26). Furthermore, in that study, a group by time interaction was revealed on ratings of problem behaviors and social initiation, suggesting a modest benefit for the AAI group over the non-AAI group. For the present study, stratified Wilcoxon Signed-Rank Tests (SAS NPAR1WAY procedure) revealed that children's self-reported scores of their behavioral conduct, scholastic and social competence were significantly higher at post-treatment than at pre-treatment in the AAI group (*z* = 2.320, *p* = .021, *z* = 2.631, *p* = .008, and *z* = 2.541, *p* = .011, respectively). Pre-post-treatment differences were not found for the children in the group without AAI.

### Correlational analyses

Post-intervention analysis in the full sample revealed no significant correlations of cognitive measures and self-esteem subscales, with the exception of children's level of math achievement as measured by the PERM-P, correlating with self-ratings of scholastic competence as measured by the SPPC (*r* = .192, *p* = .046). This small magnitude correlation did not reach significance in either subgroup (AAI *r* = .218, *p* = .110; Non-AAI *r* = .157, *p* = .267).

Likewise, for the whole sample post-intervention, behavioral measures of ADHD symptoms were not correlated with self-esteem (i.e., scores on the SPPC). Parent-reported symptoms of ODD, however, were positively correlated with child reports in the domains of Scholastic Competence (*r* = .215, *p* = .044), Athletic Competence (*r* = .307, *p* = .004), and Physical Appearance (*r* = .214, *p* = .045). That is, greater impairment from ODD symptoms as rated by the parent was linked to children more favorably perceiving their competence in these domains.

When stratified by group, however, no significant relationships between parent ratings of ODD symptoms and self-esteem were revealed in the AAI group. The finding persisted in the non-AAI group: higher parent ratings of ODD symptoms were related to greater self-perceptions of Scholastic Competence (*r* = .366, *p* = .016), Athletic Competence (*r* = .601, *p* < 0.001), and Physical Appearance (*r* = .361, *p* = .017), and Social Competence (*r* = .340, *p* = .026). Furthermore, when stratified, parent ratings of H/I were positively correlated with Scholastic Competence (*r* = .276, *p* = .033).

## Discussion

The specific aim of the present study was to determine if AAI improved self-esteem in children with ADHD when compared to more traditional psychosocial interventions. Findings from the present study indicate children's perceptions about their social competence, behavioral conduct and scholastic competence, were significantly higher at post-intervention when compared to pre-intervention in the group in which live animals participated (AAI). Conversely, no pre-post-intervention differences in self-perceptions were found for the children who participated in the intervention without dogs (non-AAI).

### Dogs and character development

Leaders in the field of HAI have called for more controlled research design with more specific aims in efforts to better understand the role of AAI in psychosocial outcomes, including self-esteem. In response, the present study employed a randomized and controlled trial of AAI vs. a “best practice” control condition with a specific aim of investigating children's self-esteem as measured by self-perceived social competence, behavioral conduct, and global self-worth. Central to the development of the treatment protocol was the consideration that elements of humane education about animals is thought to contribute to character development, social communication, and compassion—all thought to be key in the treatment aims for children with ADHD who present with deficits in skills of executive function necessary for self-regulation and self-awareness. The authors proposed that utilizing these educational strategies would be enhanced by the presence of a live animal and the integration of canine assisted intervention activities. Group differences in these findings provide support for the participation of dogs in intervention strategies aimed to improve key elements of self-esteem.

### The role of feedback from a live animal and self-regulation

Children who participated in AAI gained better access to aspects of the intervention, specifically targets of social competence and behavioral conduct. Pre-intervention, all children rated themselves about average across domains when compared with published norms. But in the areas in which they rated themselves a bit lower (social competence and behavioral conduct; presumed weaknesses in this population and specific targets of the intervention) only children in the AAI group rated themselves more favorably post-intervention. Direct interaction with a live animal provides immediate feedback of socially appropriate and compassionate behavior toward the dogs. Results suggest the tailored humane education in the session content, coupled with interaction with live dogs, served as an immediate and non-verbal means of feedback to which the child responded positively when compared to their peers in the non-AAI group.

### The role of parent and child engagement

As previously reported, parent ratings indicated that all children in the sample responded favorably on aims of symptom reduction and improved social skills, with the children in the AAI group making the strongest gains (26). The behavioral parent training (BPT) component for both intervention groups incorporated parent/child “homework” around human education, character development, and compassion. It is likely that the increased use of positive parenting strategies signals to the child that he/she is doing better. As such, the child in turn rate himself or herself better. But this supposition should have held true across both groups as all parents participated in the same BPT. Taken together, the findings suggest that the families in the AAI group responded differentially to the lessons of the BPT.

The research suggests that as a consequence of experiencing direct contact with therapy dogs, children were more motivated to attend and positively engage in the intervention. Several studies have found that incorporating therapy animals into activities can help motivate children to comply with the therapeutic or educational process, and to retain that motivation over time (42–46). In P.A.C.K. sessions, children were “frontloaded” and practiced aspects of the parent/child interaction assignment for that week in the session prior to the parent receiving the “homework.” It seems plausible that children who “role-played” with live animals, more readily recalled or shared elements of the lesson more vividly with their parents about how they acted and behave in their presence.

One might also posit that parents of children who participated in the group with therapy dogs found it easier to engage in discussion about the intervention sessions by talking about the animals instead of the content alone. By doing so, they may have directly promoted stronger generalization of the strategies learned by the children in session. It is plausible then to suspect that those parents began to notice improvement in their child's social skills and behavioral conduct more readily and thereby were more likely to express praise and appreciation at increased frequency when compared to parents of children in the non-AAI group. When parents perceive and report behavioral improvement, it is likely that children in turn receive this feedback, directly or indirectly, reinforcing their perceptions of their improvements and increasing their perceptions of their social competence and behavioral conduct.

### Limitations and future investigation

#### Positive illusory bias

Self-perception is a construct largely influenced by psychosocial development with typical younger children presenting with a lack self-awareness marked by inflated self-perceptions when compared to older children (32). Theoretically, children with neurodevelopmental disorders marked by deficits in EF may be delayed in the development of this skill when compared to their typically developing peers. The severity of specific cognitive deficits in children with ADHD are associated with positive illusory bias (47) and particularly self-awareness (48). It may be that some children with ADHD are relatively delayed in their ability to attend to, respond to, or benefit from feedback from peers, teachers or caregivers commensurate to their peers. In fact, despite frequent feedback from peers, teachers and caregivers, these children oftentimes demonstrate poor self-awareness of their problems and rate themselves more favorably than their teachers and parents rate their actual performance in academic and social context (49). This phenomenon, positive illusory bias, among children with ADHD has been well described (23, 49–51). Furthermore, children with ADHD who also demonstrate positive illusory bias are found to demonstrate greater social impairment when compared to their typically developing peers and their peers with ADHD who do not demonstrate positive illusory bias (52)

Alternative hypotheses have been presented about the nature of positive illusory bias. Some propose an over-estimation of one's competences acts as a protective mechanism for children who feel bad or embarrassed about their problem behaviors and or social challenges or that denial of weaknesses is a defense mechanism (53–55). More important, perhaps, is the finding that the degree of over-estimation of confidence also seems to mediate poor response to feedback (56). This lack of response to feedback is especially relevant to developing interventions for this population.

In the present study, the non-normal distribution of the children's self-perception scores across each domain has implications for interpreting improved scores at post-intervention for the AAI group. The majority of children in the present study, similar to their typically developing, same age peers, rated their competence fairly favorably. This finding is not surprising given the literature on the influence of development on self-perception and self-awareness. In fact, considering children with ADHD have been found to be relatively delayed in their ability to respond to social feedback and demonstrate susceptibility to positive illusory bias, one might hypothesize that these children would have rated themselves significantly more positively than their same age, typically developing peers. But in the present study, participating children seemed to perceive their competence about the same as their typically developing peers across each domain.

While not significantly different from norms reported for typically developing children of similar age, the scores indicate that the children in the present study may not accurately perceive their competence when compared to how their parents and peers perceive them. While group differences in increased social, and scholastic competence and behavioral conduct may be a function of greater parent reported improvement, these increases do not necessarily mean that children demonstrated behavior commensurate with their self-estimations.

This finding may suggest a continued lack of self-awareness despite improvement and increased self-regard. Alternatively, it may be simply a function of their age compounded by their diagnosis of a neurodevelopmental delay. Conversely, it may simply mean those in the AAI group felt better about themselves and they reported so. Further investigation of this phenomenon among children participating in AAI and the control condition is indicated and should include an analysis of difference scores between parent/teacher and peer ratings of competence and children's self-ratings of competence to more directly measure the possibility of positive illusory bias in this group. Additional considerations include an examination of the associations between improved self-perceptions of competence and behavioral conduct with baseline reports of externalizing behaviors, observed compliance and self-regulation in the course of psychosocial intervention with and without dogs.

#### Targeting self-esteem and measuring it as an outcome

Brummelman addressed contemporary criticism that interventions aimed at increasing self-esteem inadvertently cultivate narcissism among children and clarified the distinction between the two constructs as “the belief that one is superior to others vs. the belief that one is worthy” (p. 11) (57). Indeed, in the AAI group, promotion of cross-species value and worthiness were reinforced with intervention activities, adult modeling, and by the positive attention directed to and received from the therapy dogs. Nevertheless, whereas self-esteem has been found to be variable and dependent on outcomes of day-to-day experiences, self-compassion is a relatively consistent way of relating to oneself that is characterized by self-kindness, a sense of common humanity, and mindfulness when encountering a personal challenge (58). Furthermore, moderating factors including aggression and internalizing symptoms may moderate ratings of self-esteem (59). Future work extending the findings of this study should more closely examine internalizing symptoms and focus on self-compassion as an alternative to self-esteem. Neff and Vonk assert that self-compassion may be an “important source of positive self-regard that is …less ego reactive and inflated” (p. 44) (58) As such, children with ADHD who encounter day-to-day challenges across contexts may benefit from explicit cultivation of self-compassion as an adaptive coping strategy that fosters well-being and resilience.

#### Medication naïve sample

To our knowledge, this work is the first randomized controlled trial examining the effectiveness of AAI with a large sample of young children with ADHD. Initial findings provide promise for increasing the modalities of treatment available to this population, but replication of these early findings is called for. Additionally, while many families seek alternatives to pharmacological interventions, evidence for the effectiveness of stimulant medications for reducing symptoms of ADHD is robust. The prescription of stimulants remains the most common practice for treating ADHD in the United States (60) and remains among the main practice parameters of the American Academy of Child and Adolescent Psychiatry (61) and the American Academy of Pediatrics (62). Of note, this study was limited to young children who were medication naïve and whose parents were seeking a non-pharmacological intervention prior to trying stimulant medications. While it was not the aim of this study to determine the effectiveness of AAI compared to stimulant medications for improving outcomes for children with ADHD, that is a viable research question that may warrant future investigation.

## Conclusion

When compared to traditional, “best practice” psychosocial intervention, psychosocial intervention with the assistance of therapy dogs (AAI) yielded greater improvements in perceived self-competence, behavioral conduct and academic competence among children with ADHD. The findings may lead future researchers to continue investigating what elements of AAI appear to impact self-esteem and how the impact can be sustained over time. AAI is becoming a more recognized complementary therapy and with more scientific support, it may become more naturally applied in numerous treatment programs serving not only children with ADHD but other children with neurodevelopmental disorders.

## Author contributions

SS was the principal investigator and led the development, interpretation, writing and dissemination of this work. HJ participated in the preparation of the data and contributed to the writing of the manuscript. MA participated in the development and implementation of the intervention, the interpretation and the writing of this manuscript. AS provided the statistical consultation and analyses of the data. AF significantly contributed to the development of the AAI content and strategies and participated in the interpretation of the findings. KL contributed to the interpretation of the results and writing of the manuscript.

### Conflict of interest statement

The authors declare that the research was conducted in the absence of any commercial or financial relationships that could be construed as a potential conflict of interest.
